# Immune dysfunctions affecting bone marrow Vγ9Vδ2 T cells in multiple myeloma: Role of immune checkpoints and disease status

**DOI:** 10.3389/fimmu.2022.1073227

**Published:** 2022-12-20

**Authors:** Claudia Giannotta, Barbara Castella, Ezio Tripoli, Daniele Grimaldi, Ilaria Avonto, Mattia D’Agostino, Alessandra Larocca, Joanna Kopecka, Mariella Grasso, Chiara Riganti, Massimo Massaia

**Affiliations:** ^1^ Laboratorio di Immunologia dei Tumori del Sangue (LITS), Centro Interdipartimentale di Biotecnologie Molecolari “Guido Tarone”, Dipartimento di Biotecnologie Molecolari e Scienze della Salute, Università degli Studi di Torino, Torino, Italy; ^2^ Struttura Complessa (SC) Ematologia, Azienda Ospedaliera (AO) S.Croce e Carle, Cuneo, Italy; ^3^ Servizio Interdipartimentale di Immunoematologia e Medicina Trasfusionale, Azienda Ospedaliera (AO) S.Croce e Carle, Cuneo, Italy; ^4^ Struttura Complessa (SC) Ematologia, Azienda Ospedaliero-Universitaria (AOU) Città della Salute e della Scienza di Torino, Torino, Italy; ^5^ Dipartimento di Oncologia, Università degli Studi di Torino, Torino, Italy

**Keywords:** Vγ9Vδ2 T cells, immune checkpoints (ICP), tumor microenvironment, multiple myeloma, chronic exhaustion, immune senescence

## Abstract

**Introduction:**

Bone marrow (BM) Vγ9Vδ2 T cells are intrinsically predisposed to sense the immune fitness of the tumor microenvironment (TME) in multiple myeloma (MM) and monoclonal gammopathy of undetermined significance (MGUS).

**Methods:**

In this work, we have used BM Vγ9Vδ2 T cells to interrogate the role of the immune checkpoint/immune checkpoint-ligand (ICP/ICP-L) network in the immune suppressive TME of MM patients.

**Results:**

PD-1+ BM MM Vγ9Vδ2 T cells combine phenotypic, functional, and TCR-associated alterations consistent with chronic exhaustion and immune senescence. When challenged by zoledronic acid (ZA) as a surrogate assay to interrogate the reactivity to their natural ligands, BM MM Vγ9Vδ2 T cells further up-regulate PD-1 and TIM-3 and worsen TCR-associated alterations. BM MM Vγ9Vδ2 T cells up-regulate TIM-3 after stimulation with ZA in combination with αPD-1, whereas PD-1 is not up-regulated after ZA stimulation with αTIM-3, indicating a hierarchical regulation of inducible ICP expression. Dual αPD-1/αTIM-3 blockade improves the immune functions of BM Vγ9Vδ2 T cells in MM at diagnosis (MM-dia), whereas single PD-1 blockade is sufficient to rescue BM Vγ9Vδ2 T cells in MM in remission (MM-rem). By contrast, ZA stimulation induces LAG-3 up-regulation in BM Vγ9Vδ2 T cells from MM in relapse (MM-rel) and dual PD-1/LAG-3 blockade is the most effective combination in this setting.

**Discussion:**

These data indicate that: 1) inappropriate immune interventions can exacerbate Vγ9Vδ2 T-cell dysfunction 2) ICP blockade should be tailored to the disease status to get the most of its beneficial effect.

## Introduction

The discovery of immune checkpoints (ICP) and their role as therapeutic targets has revitalized immunotherapy in cancer (1). However, clinical results have been discontinuous with major achievements in some diseases and negligible or disappointing results in others (2–5). Both primary and acquired resistance have been reported to hamper the efficacy of ICP blockade, but the underlying mechanisms have only partially been elucidated. Multiple myeloma (MM) is a paradigm disease in which the immune system and the tumor microenvironment (TME) play a major role in disease progression (6–8). Several phenotypic and functional alterations have been reported in innate and adaptive immune effector cells, including the expression of ICP/ICP ligands in myeloma cells and bystander cells in the TME (9–11). Despite these favourable premises, single αPD-1 treatment has fallen short of clinical expectations in MM, whereas clinical studies of αPD-1 in combination with immunomodulatory drugs (IMiDs) have been terminated ahead of time because of unexpected toxicity in the experimental arm. These unsuccessfully immune interventions have led to the premature termination of alternative studies targeting the ICP/ICP-L network and generated some reluctance in further pursuing this approach due to the complexity of the tumor-host interactions in MM (12).

Vγ9Vδ2 T cells from the bone marrow (BM) are excellent tools to monitor the immune suppressive commitment and decode the ICP/ICP-L network in MM patients (13). Vγ9Vδ2 T-cells are non-conventional T cells half-way between adaptive and innate immunity with a natural inclination to react against malignant B cells, including myeloma cells (14). This intrinsic susceptibility is due to the enhanced cell surface expression of stress-induced self-ligands and the intense production of phosphorylated metabolites generated by the mevalonate (Mev) pathway (14). Isopentenyl pyrophosphate (IPP) is the prototypic Mev metabolite recognized by Vγ9Vδ2 T cells *via* the combination of two immunoglobulin superfamily members, butyrophilin 2A1 (BTN2A1) and BTN3A1. The former directly binds the Vγ9+ domain of the T cell receptor (TCR), whereas the latter binds the Vδ2 and γ-chain regions on the opposite side of the TCR (15–18). IPP is structurally related to the phosphoantigens (pAgs) generated by bacteria and stressed cells that are patrolled by Vγ9Vδ2 T cells as part of their duty to act as first-line defenders against infections and stressed cell at risk of malignant transformation (19). By interrogating the reactivity of BM MM Vγ9Vδ2 T cells to IPP generated by monocytes or dendritic cells (DC) after stimulation with zoledronic acid (ZA), we have revealed a very early and long-lasting dysfunction of BM Vγ9Vδ2 T cells which is already detectable in monoclonal gammopathy of undetermined significance (MGUS) and not fully reverted in clinical remission after autologous stem cell transplantation (9). Multiple cell subsets [myeloma cells, myeloid-derived suppressor cells (MDSC), regulatory T cells (Tregs), BM-derived stromal cells (BMSC)] are involved in Vγ9Vδ2 T-cell inhibition *via* several immune suppressive mechanisms including PD-1/PD-L1 expression (9, 10). Previous work from our lab has shown that single PD-1 blockade improved ZA-induced proliferation of BM MM Vγ9Vδ2 T cells from MM at diagnosis (MM-dia). PD-1 blockade also increased CD107 expression suggesting improved effector functions, but both proliferation and CD107 expression remained far from standard values observed in BM Vγ9Vδ2 T cells from controls (Ctrl) (9).

Recently, it has been reported that the expression of additional ICP on immune effector cells can be involved in acquired resistance to single ICP blockade. PD-1 and TIM-3 co-expression has been reported in conventional T cells from patients with solid cancers (20–23), AML (24), and MM (25–27). PD-1 and TIM-3 co-expression has also been reported in Vγ9Vδ2 T cells chronically exposed to infectious agents (28) or to cancer cells in solid (29, 30) and blood tumors (31). Exhaustion and immune senescence are other T-cell dysfunctions which can potentially contribute to resistance to ICP blockade (32–35).

The aim of this work was to investigate the contribution of ICP expression, exhaustion, and immune senescence to the dysfunction of BM MM Vγ9Vδ2T cells and to envisage possible interventions, correlated with the disease status, to overcome the immune suppressive commitment operated by the ICP/ICP-L network in the TME of MM patients.

## Methods

### Samples collection

Bone marrow mononuclear cells (BMMC) from BM aspirates and autologous peripheral blood mononuclear cells (PBMC) from MM patients at different stages of disease (diagnosis: MM-dia; remission: MM-rem; relapse: MM-rel) were used for the study. All experiments were performed with BM samples from MM-dia unless otherwise specified. BMMC from patients with hematological malignancies in unmaintained molecular remission, frozen human normal BMMC purchased from Stem Cell Technologies, and PBMC from healthy donors attending the local Blood Bank were used as control (Ctrl). The study was approved by institutional regulatory boards (n.176-19 December 11, 2019).

### Cell surface and intracellular flow cytometry

The monoclonal antibodies (mAbs) used in the study are listed in **Supplemental Table I**. Cell surface and intracellular flow cytometry were performed as previously reported (9). Vγ9Vδ2 T cells were identified with αTCR Vγ9 mAb conjugated with the appropriate fluorochrome (FITC, PE, APC) depending on the multicolor staining combination (see **Supplemental Table I**). We have intentionally focused on Vγ9Vδ2 T cells because this is the only γδ T-cell subset directly activated by pAgs or indirectly activated by ZA stimulation (36–38). Moreover, Vδ2 chain is the only one to combine with the Vγ9 chain confirming that αTCR Vγ9 mAbs are reliable tools to identify Vγ9Vδ2 T cells (39). Cytofluorimetric analyses were performed with FACS Calibur Cell Sorter and FlowJo software (Becton Dickinson, Mountain View, CA).

### Vγ9Vδ2 T-cell proliferation, cytokine release and degranulation

Cryopreserved or freshly isolated PBMC or BMMC from MM patients and Ctrl were cultured for 7 days with 10 IU/ml IL-2, and 1 µM ZA+10 IU/ml IL2. In selected experiments, cells were cultured in the presence of αPD1 (10 μg/ml), αTIM-3 (10 μg/ml), αLAG-3 (10 μg/ml), or a combination thereof. Proliferation was evaluated by calculating total counts of viable Vγ9Vδ2 T cells on day 7 with the trypan blue staining assay and flow cytometry after gating for CD3 in combination with appropriate αVγ9 mAb. IL-17 production was evaluated in freshly isolated BMMC after incubation with PMA (50 ng/ml)/Ionomycin (1 μg/ml) for 4 hours at 37°C and 5% CO2 with brefeldin (500 ng/ml) added during the last hour. IFN-γ, and CD107 expression were evaluated as previously reported (9).

### Conventional T- cell proliferation

Conventional T-cell proliferation was measured by carboxyfluorescein-diacetate-succinimidyl-ester (CFSE) dilution assay. BMMC were suspended in warmed PBS at a concentration of 10×10^6^ cells/ml and labeled with 1 μM CFSE at 37°C for 15 min in the dark. After quenching with FCS for 10 minutes in dark at 37°C and washing with RPMI medium, cells were seeded at 1×10^6^ cells/ml in 96‐well flat‐bottom plate and stimulated with αCD3 (1 μg/ml - BioLegend) and αCD28 (2 μg/ml - BioLegend) antibodies for 72 h at 37°C. After 3 days, conventional T cells where harvested and identified with αCD8 and αCD4 rather than αCD3 given the down-modulation induced by αCD3/αCD28 stimulation and the lineage discrimination capacity of CD4 and CD8 expression (40). In selected experiments, the proliferation of BM CD4 and CD8 T cells with αCD3 and αCD28 was performed in the presence (BMMC) or absence of γδ T cells (BMMC-γδ-). Depletion was performed by immune magnetic separation using Anti-pan-γδ-conjugated magnetic microbeads (Miltenyi Biotec, Germany #130-050-701).

### Western blots

For Western blot experiments, γδ T cells were purified by immune magnetic separation using Anti-pan-γδ-conjugated magnetic microbeads (Miltenyi Biotec, Germany #130-050-701). Purity was always > 90% by FITC-conjugated-Hapten MicroBeads staining (Miltenyi Biotec, Germany #130-050-701). After ZA stimulation, Vγ9Vδ2 T cells were the predominant population also in MM patients who did not respond to ZA stimulation (**Supplemental Figure 1**). Cells were lysed in a MLB buffer (125 mM Tris-HCl, 750 mM NaCl, 1% v/v NP40, 10% v/v glycerol, 50 mM MgCl2, 5 mM EDTA, 25 mM NaF, 1 mM NaVO4, 10 μg/ml leupeptin, 10 μg/ml pepstatin, 10 μg/ml aprotinin, 1 mM phenylmethylsulphonyl fluoride, pH 7.5), sonicated and centrifuged at 13,000 × g for 10 min at 4°C. Twenty μg of proteins from cell lysates were subjected to Western blotting and probed with the antibodies listed in **Supplemental Table II**. The proteins were detected by enhanced chemiluminescence (Bio-Rad Laboratories). The band density analysis was performed using the ImageJ software (https://imagej.nih.gov/ij/) and expressed as arbitrary units. The ratio band density of each protein/band density of tubulin (as housekeeping protein) was calculated in each experimental condition. For untreated/baseline/unstimulated cells, the band density ratio was considered 1. For the other experimental conditions, the ratio was expressed as proportion towards the ratio obtained in untreated cells.

### ELISA

Supernatants (S/N) from Ctrl and MM BMMC stimulated for 7 days with 10 IU/ml IL2, 1 µM ZA+10 IU/ml IL2 in the presence or absence of αPD1 were collected and stored at -80°C. The concentration of human IL27 was quantified in S/N by enzyme-linked immunosorbent assay (ELISA) technology with the IL-27 Human ELISA kit (Invitrogen; Catalogue number: # BMS2085) according to manufacturer’s instructions.

### Statistical analysis

The results are expressed as mean ± SE. Differences between the groups have been evaluated with the one-way analysis of variance, and the Wilcoxon–Mann–Whitney non-parametric test for paired or unpaired samples as appropriate and considered to be statistically significant for p values <0.05. Correlation analyses have been performed with the non-parametric Spearman Rank Order test with a cut-off p value <0.05.

## Results

### Dual PD-1/TIM-3 expression, functional exhaustion, and immune senescence are intertwined in BM MM Vγ9Vδ2 T cells

**Figure 1:**
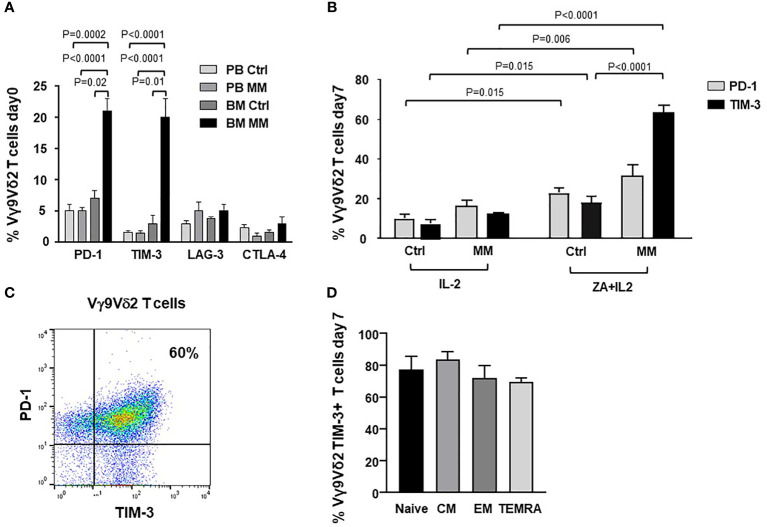
ICP expression and subset distribution in resting and ZA-stimulated BM MM Vγ9Vδ2 T cells. **(A)** PD-1, TIM-3, LAG-3 and CTLA-4 expression in resting PB and BM Vγ9Vδ2 T cells from healthy subjects (Ctrl) and MM at diagnosis. Bars represent mean values ± SE from 5 (BM Ctrl) to 30 (BM MM) experiments. **(B)** PD-1 and TIM-3 expression are significantly increased after ZA stimulation in MM BM Vγ9Vδ2 T cells. Bars represent mean values ± SE from 7 (BM Ctrl) to 30 (BM MM) experiments; **(C)** Cytofluorimetric analysis of PD-1 and TIM-3 co-expression in BM MM Vγ9Vδ2 T cells from one representative MM after ZA stimulation. **(D)** TIM-3 expression in naive (CD27+ CD45RA+), central memory (CM) (CD27+ CD45RA-), effector memory (EM) (CD27- CD45RA-), and terminally differentiated effector memory (TEMRA) (CD27- CD45RA+) BM MM Vγ9Vδ2 T cells after ZA stimulation. CM BM MM Vγ9Vδ2 T cells show the highest TIM-3 expression. Bars represent mean values ± SE of 3 experiments.

**Figure 1A** shows PD-1, TIM-3, LAG-3 and CTLA-4 expression in resting PB and BM Vγ9Vδ2 T cells from Ctrl and MM patients. PD-1 and TIM-3 expression was significantly higher in BM of MM patients than in Ctrl samples. After ZA stimulation, BM MM Vγ9Vγ2 T cells further increased PD-1 (9) and TIM-3 expression (**Figure 1B**), while the increase in BM Ctrl Vγ9Vδ2 T cells was limited and significantly lower (**Figure 1B**). Cytofluorometric analysis from one representative MM shows that PD-1 and TIM-3 are co-expressed by approximately 60% of BM MM Vγ9Vδ2 T cells after ZA stimulation (**Figure 1C**). In freshly isolated Vγ9Vδ2 T cells we have previously shown that central memory (CM) Vγ9Vδ2 T cells display the highest PD-1 expression (9). After ZA stimulation, TIM-3 up-regulation was documented in all Vγ9Vδ2 T-cell subsets with CM and naïve Vγ9Vδ2 T cells showing slightly higher levels than effector memory (EM) and terminally differentiated effector memory (TEMRA) Vγ9Vδ2 T cells (**Figure 1D**). The gating strategy used to investigate TIM-3 expression in Vγ9Vδ2 T-cell subsets is shown in **Supplemental Figure 2**.

**Figure 2:**
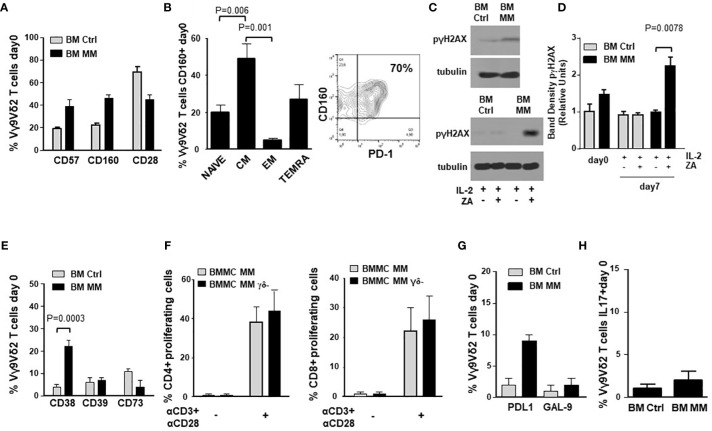
ICP expression in BM MM Vγ9Vδ2 T cells is associated with chronic exhaustion and immune senescence markers. **(A)** CD57, CD160 and CD28 expression in BM Ctrl and BM MM Vγ9Vδ2 T cells. Bars represent mean values ± SE from 3 (BM Ctrl) to 50 (BM MM) experiments. Differences are not statistically significant. **(B)** *left:* CM is the BM MM Vγ9Vδ2 T-cell subset with the highest CD160 expression. Bars represent mean values ± SE of 8 experiments; *right:* cytofluorometric analysis of CD160 and PD-1 co-expression in BM MM Vγ9Vδ2 T cells from one representative sample. **(C)** Western blot analysis of p-γH2AX expression in resting (upper panel) and ZA-stimulated (lower panel) Vγ9Vδ2 T cells from one representative BM Ctrl and MM sample. Tubulin expression is shown to confirm equal protein loading per lane. **(D)** Densitometric analysis of pooled p-γH2AX expression data in resting (day 0) and ZA-stimulated (day 7) BM Ctrl and MM Vγ9Vδ2 T cells. Bars represent mean values ± SE from 1 (BM Ctrl and BM MM d0) to 4 (BM MM) experiments. **(E)** CD38, CD39, and CD73 expression in resting BM Ctrl and BM MM Vγ9Vδ2 T cells. Bars represent mean values ± SE from 8 (BM Ctrl) to 40 (BM MM) experiments. **(F)** CFSE-based analysis of BM MM CD4+ and CD8+ proliferation after 72-hour stimulation with αCD3 + αCD28 in the presence (BMMC) or absence (BMMC- γδ- T-cell depleted) of BM Vγ9Vδ2 T cells. Bars represent mean values ± SE of 3 experiments. **(G)** PD-L1 and GAL-9 expression in resting BM Ctrl and MM Vγ9Vδ2 T cells. Bars represent mean values ± SE from 4 (BM Ctrl) to 16 (BM MM) experiments. **(H)** Intracellular IL-17 expression in resting BM Ctrl and MM Vγ9Vδ2 T cells after PMA+ ionomycin stimulation. Bars represent mean values ± SE from 3 (BM Ctrl) to 15 (BM MM) experiments.

**Figure 2A** compares the expression of immune senescence markers (33, 41, 42) in BM Ctrl and MM Vγ9Vδ2 T cells. BM MM Vγ9Vδ2 T cells showed significantly higher CD57 and CD160, and lower CD28 expression than BM Ctrl Vγ9Vδ2 T cells, even if differences were not statistically significant. The highest CD160 expression was observed in CM BM Vγ9Vδ2 T cells which is the cell subset with the highest PD-1 (9) and TIM-3 expression (**Figure 2B**). Cytofluorometric analysis of CD160 and PD-1 co-expression in BM MM Vγ9Vδ2 T cells from one representative sample is shown in **Figure 2B** (right panel).

Phosphorylated-γH2AX (p-γH2AX) is an early marker of DNA damage associated to immune senescence (43). p-γH2AX expression in BM Ctrl and MM-dia Vγ9Vδ2 T cells is shown in **Figure 2C** (one representative experiment) and **Figure 2D** (pooled data). These experiments were performed on purified γδ T cells. Both Vδ1 and Vγ9Vδ2 subsets can be represented in variable proportions in freshly purified γδ T cells (day 0), whereas after ZA stimulation Vγ9Vδ2 T cells become predominant (**Supplemental Figure 1**) and any change should be referred to these because they are the only γδ T-cell subset sensitive to ZA stimulation. In freshly isolated BM γδ T cells, p-γH2AX expression was slightly higher in MM than Ctrl, but the difference was not statistically significant. After ZA stimulation, p-γH2AX expression was significantly increased in BM MM only (**Figures 2C, D**).

IL-7 has been reported to mitigate the induction of immune senescence of conventional T cells exposed to tumor cells (44, 45). We have investigated whether exogenous IL-7 could relieve the immune dysfunction of BM MM Vγ9Vδ2 T cells, but we have not observed any beneficial effect (data not shown).

Accumulating evidences indicate that Vγ9Vδ2 T cells can exert different functions depending on the local microenvironment, including the ability to promote tumor progression *via* the acquisition of regulatory or pro-tumoral functions (46). **Figure 2E** shows the expression of CD38, CD39, and CD73 in BM Vγ9Vδ2 T cells from Ctrl and MM patients. These molecules cooperate in the induction of the immune suppressive TME in MM *via* adenosine production (47). Only CD38 was significantly up-regulated in MM compared with Ctrl, whereas no differences were observed in CD39 and CD73 expression. The adenosine circuitry operated by CD38, CD39, and CD73 is well known to contribute to the establishment of the immune suppressive contexture in the TME of MM (47), but our data indicate that Vγ9Vδ2 T cells are not directly involved in this immune suppressive circuitry.

Lastly, BM MM Vγ9Vδ2 T cells did not show any phenotypic and/or functional features consistent with suppressor and/or pro-tumoral functions. The proliferation of CD4+ and CD8+ T cells after αCD3/αCD28 stimulation was similar in the presence or absence of γδ T cells (**Figure 2F**). **Supplementary Figure 3A** shows that proliferation of BM MM CD4+ and CD8+ cells was similar or even better compared with PB Ctrl CD4+ and CD8+ cells. Unlike BM Vγ9Vδ2 T cells, CD4+ and CD8+ cell proliferation was not influenced by the disease status (**Supplementary Figure 3B**), confirming the unique BM MM Vγ9Vδ2 T-cell susceptibility to the immune suppressive TME contexture.

The expression of PD-L1, GAL-9 and IL-17 characterizes Vγ9Vδ2 T cells with pro-tumoral functions in the TME (48). As shown in **Figures 2G, H**, the expression of GAL-9 and cytoplasmic IL-17 was similar in BM Ctrl and MM Vγ9Vδ2 T cells except for PD-L1 expression, which was slightly increased in the former, but the difference was not statistically significant. Representative dot plots of IL-17 expression in BM MM and Ctrl Vγ9Vδ2 T cells are shown in **Supplemental Figure 4**.

### Altered expression of TCR-associated molecules in BM MM Vγ9Vδ2 T cells

**Figure 3:**
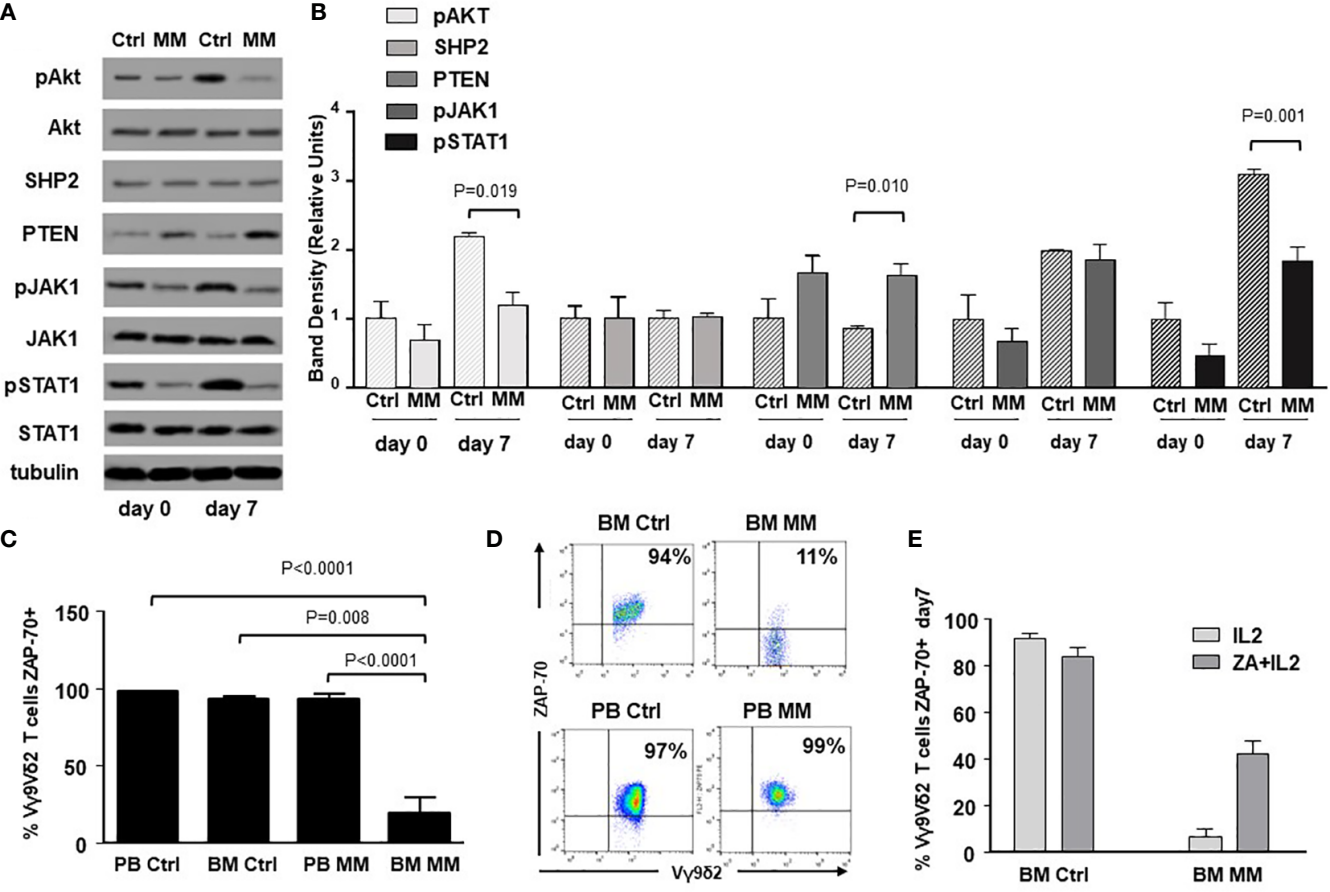
Alterations of TCR-associated molecules in BM MM Vγ9Vδ2 T cells. **(A)** Western blot analysis of selected TCR-associated molecules (pAKT, AKT, SHP2, PTEN, pJAK-1, JAK-1, pSTAT-1, STAT-1) in purified resting (day 0) and ZA-stimulated (day 7) BM γδ T cells from one representative Ctrl and MM. pAKT, pJAK and pSTAT-1 are down-regulated, whereas PTEN is up-regulated in resting BM MM γδ T cells. These differences are amplified after ZA stimulation (day 7). Tubulin expression is shown to confirm equal protein loading per lane. **(B)** Densitometric analysis of pooled data from ZA-stimulated BM Ctrl and BM MM γδ T cells confirms lower expression of pAKT, and pSTAT1, and higher PTEN expression in BM MM γδ T cells *vs* BM Ctrl γδ T cells. Bars represent mean values ± SE from 1 (BM Ctrl d0 and BM MM d0) to 14 experiments (BM MM). **(C)** ZAP-70 expression in resting PB and BM Vγ9Vδ2 T cells from Ctrl and MM patients. Bars represent mean values ± SE from 3 (BM Ctrl) to 25 experiments (BM MM); **(D)** cytofluorimetric analysis of ZAP-70 expression in Vγ9Vδ2 T cells from BM and PB MM Vγ9Vδ2 T cells and BM and PB Ctrl; **(E)** ZAP-70 expression after ZA stimulation in Ctrl and MM BM Vγ9Vδ2T cells. Bars represent mean values ± SE from 2 (BM Ctrl) to 3 experiments (BM MM).

ICP expression and immune senescence in T cells are associated with defective intracellular TCR signaling (49, 50). **Figure 3A** shows the expression of selected TCR-associated molecules in purified BM γδ T cells from one representative Ctrl and MM patient on day 0 and after ZA-stimulation (day 7). As reported above, both Vδ1 and Vγ9Vδ2 cells are represented in freshly purified γδ T cells (day 0), whereas Vγ9Vδ2 T cells are predominant on day 7 and they are the only γδ T-cell subset engaged by ZA (**Supplemental Figure 1**). Pooled data are shown in **Figure 3B** showing that BM MM Vγ9Vδ2 T cells had signiﬁcantly lower pAKT, higher PTEN, and lower pSTAT-1 expression on day 7 compared to BM Ctrl Vγ9Vδ2 T cells.

ZAP-70 and CD3-ζ chain are other TCR-associated molecules defectively expressed in T cells from the TME of mice and humans (51). ZAP-70 expression was significantly lower in resting BM MM Vγ9Vδ2 T cells compared with PB and BM Ctrl Vγ9Vδ2 T cells, but also with PB MM Vγ9Vδ2 T cells (**Figure 3C**), further confirming the striking difference between circulating *vs* TME-resident Vγ9Vδ2 T cells. Representative dot plots are shown in **Figure 3D**. Paired analyses of Vγ9Vδ2+ and CD3+ Vγ9Vδ2- cells showed that the mean ZAP-70 expression was also significantly down-regulated in BM CD3+ Vγ9Vδ2- T cells of MM patients with a wide range of expression in individual samples (**Supplemental Figure 5**). A slight increase was observed after ZA stimulation in Vγ9Vδ2 T cells from 3 MM patients with low ZAP-70 expression at baseline, but values remained inferior to Ctrl values (**Figure 3E**). Unlike ZAP-70, the proportion and MFI of CD3-ζ chain expression were not different in PB and BM Ctrl and MM Vγ9Vδ2 T cells (**Supplemental Figure 6**).

### PD-1/TIM-3 cross-talk in BM MM Vγ9Vδ2 T cells

**Figure 4:**
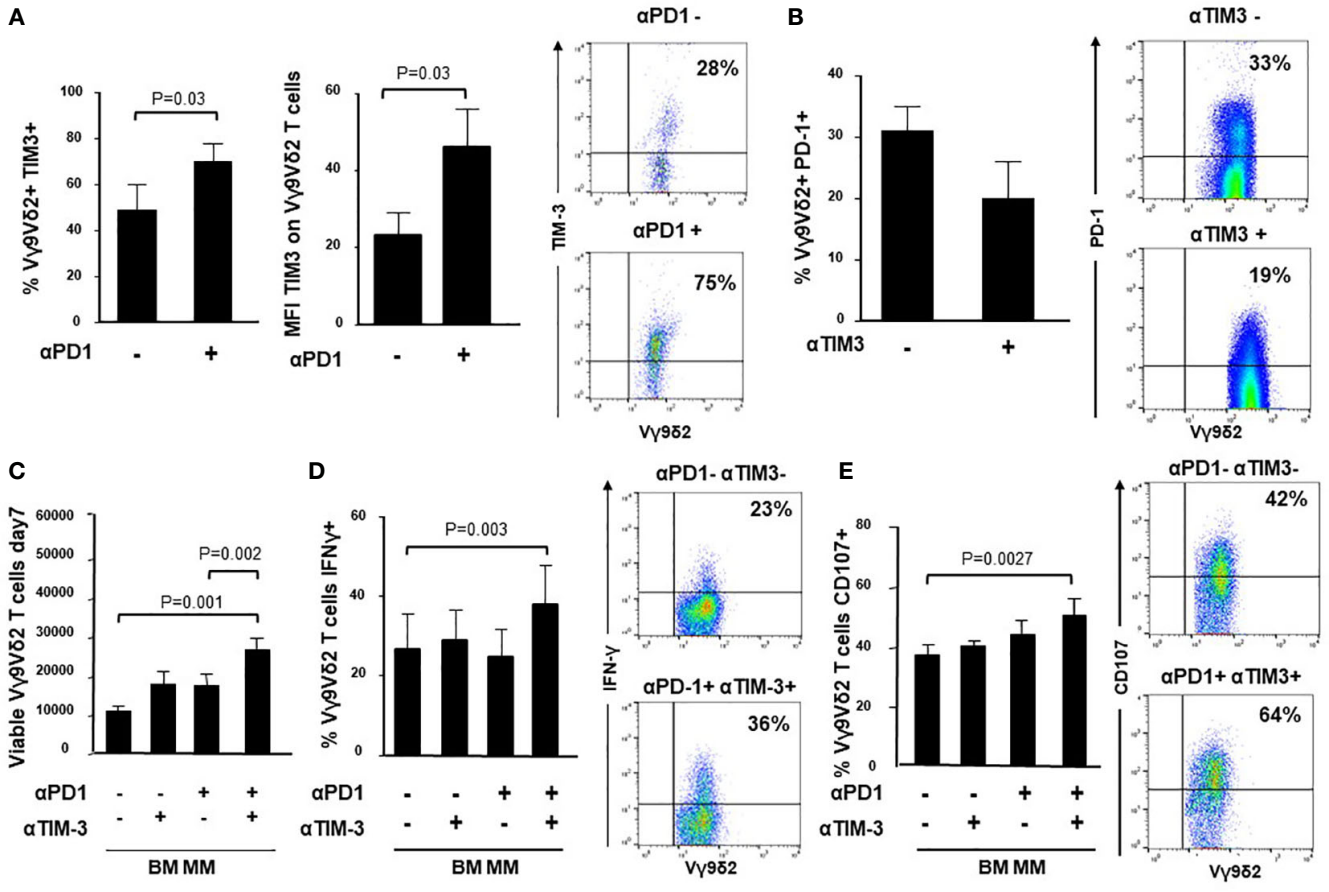
Intracellular cross-talk between PD-1 and TIM-3 in BM MM Vγ9Vδ2 T cells. **(A)** *left:* Percentage and MFI of TIM-3+ cells are significantly up-regulated in BM MM Vγ9Vδ2 T cells after ZA stimulation in the presence of αPD1. Bars represent mean values ± SE of 6 experiments; *right:* cytofluorimetric analysis of TIM-3 expression after ZA stimulation in the absence (upper panel) or in the presence (lower panel) of αPD-1 in one representative experiment; **(B)** *left:* PD-1 expression is slightly down-regulated in BM MM Vγ9Vδ2 T cells after ZA stimulation in the presence of αTIM-3, but the difference is not statistically significant. PD-1 expression is significantly up-regulated after ZA stimulation as already reported in Figure 1B. Bars represent mean values ± SE of 5 experiments; *right:* cytofluorimetric analysis of PD-1 expression after ZA stimulation in the absence (upper panel) or in the presence (lower panel) of αTIM-3 in one representative experiment; **(C)** ZA-induced BM MM Vγ9Vδ2 T-cell proliferation in the absence or in the presence of αPD-1, αTIM-3 and the combination thereof. Bars represent mean values ± SEM of 5 experiments. **(D)** *left:* intracellular IFN-γ production by ZA-stimulated BM MM Vγ9Vδ2 in the absence or in the presence of αPD-1, αTIM-3 and the combination thereof. Bars represent mean values ± SEM of 4 experiments; *right:* cytofluorimetric analyses of IFN-γ production in BM MM Vγ9Vδ2 T cells after ZA stimulation in the absence (upper panel) or in the presence (lower panel) of dual PD1/TIM-3 blockade. **(E)** *left:* CD107 expression in ZA-stimulated BM MM Vγ9Vδ2 in the absence or in the presence of αPD-1, αTIM-3, and the combination thereof. Bars represent mean values ± SE of 6 experiments; *right:* cytofluorimetric analyses of CD107 expression in BM MM Vγ9Vδ2 T cells after ZA stimulation in the absence (upper panel) or in the presence (lower panel) of dual blockade PD1/TIM-3 blockade.

It has been reported that TIM-3 up-regulation is involved in the acquired resistance to PD-1 blockade (31, 52, 53). Thus, we have investigated whether TIM-3 was involved in the incomplete recovery of BM MM Vγ9Vδ2 T cells after ZA stimulation and single PD-1 blockade. **Figure 4A** shows that both TIM-3 expression and MFI values were significantly up-regulated in BM MM Vγ9Vδ2 T cells in the presence of αPD-1, whereas PD-1 expression was slightly down-regulated after ZA stimulation in the presence of αTIM-3, but the decrease was not statistically significant. Representative cytofluorometric analyses of increased TIM-3 up-regulation and PD-1 down-regulation are shown in **Figure 4A** (right panel) and **Figure 4B** (right panel).

Next, we investigated whether dual PD1-/TIM-3 blockade was more effective than single blockade. We evaluated the proliferation (**Figure 4C**), IFN-γ production (**Figure 4D**) and CD107 expression (**Figure 4E**) in BM MM Vγ9Vδ2 T cells after ZA stimulation in the presence of αPD-1, αTIM-3, and the combination thereof. Representative cytofluorometric analyses of increased IFN-γ and CD107 expression in BM MM Vγ9Vδ2 T cells after dual blockade are shown in **Figure 4D** (right panel) and **Figure 4E** (right panel). Our results indicate that dual blockade PD-1/TIM-3 blockade is more effective than single PD-1 or TIM-3 blockade in MM-dia to mitigate BM MM Vγ9Vδ2 T-cell dysfunctions.

**Figure 5:**
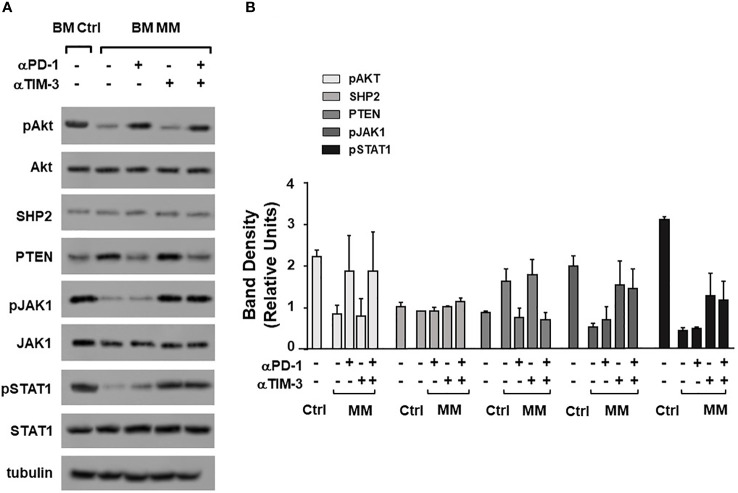
Alterations of TCR-associated molecules are mitigated by αPD-1 and/or αTIM-3. **(A)** Western blot analysis of pAKT, AKT, SHP2, PTEN, pJAK-1, JAK-1, pSTAT-1, and STAT-1 expression in BM Ctrl and BM MM γδ T cells from one representative experiment after ZA stimulation in the absence or in the presence of αPD1, αTIM-3, and the combination thereof. Tubulin expression is shown to confirm equal protein loading per lane. **(B)** Densitometric analysis of pooled data. Bars represent mean values ± SE of 2 experiments.

Dual PD-1/TIM-3 blockade was also associated with a partial recovery of TCR-associated alterations. Data from one representative Ctrl and MM are shown in **Figure 5A**, while pooled data from 2 paired experiments are shown in **Figure 5B**. αPD-1 partially normalized pAKT and PTEN expression, whereas αTIM-3 partially normalized pJAK1 and pSTAT1 expression. No antagonist, additive or synergistic effect was observed suggesting that αPD-1 and αTIM-3 target mutually exclusive TCR-associated molecules in BM MM Vγ9Vδ2 T cells. **Supplementary Figure 8** shows pooled data from unpaired experiments after αPD-1 treatment only.

### Intracellular PD-1/TIM-3 cross-talk is not mediated by the IL-27/pSTAT1/T-bet or the PI3K-AKT pathways

**Figure 6:**
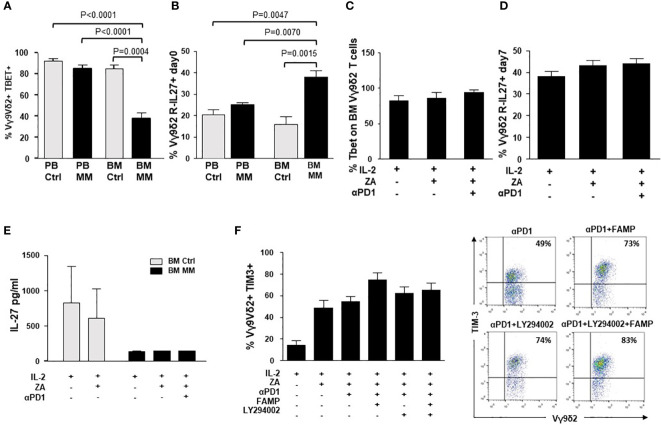
Intracellular PD-1/TIM-3 cross-talk is not mediated by the IL-27/pSTAT1/T-bet or PI3K-AKT axes. **(A)** T-bet and MFI expression in resting PB and BM Ctrl and MM Vγ9Vδ2 T cells. Bars represent the mean values ± SE from 6 (BM Ctrl) to 25 experiments (BM MM); **(B)** IL-27R expression in resting PB and BM Ctrl and MM Vγ9Vδ2 T cells. Bars represent the mean values ± SE from 6 (BM Ctrl) to 25 experiments (BM MM). **(C)** T-bet and **(D)** IL-27R expression in ZA-stimulated BM MM Vγ9Vδ2 T cells with or without αPD1. Bars represent mean values ± SE from 4 (Tbet) to 6 (IL-27R) experiments **(E)** IL-27 concentrations in the supernatants (S/N) of ZA-stimulated BMMC from Ctrl and MM patients. Bars represent the mean values ± SE from 3 (BM Ctrl) to 4 experiments (BM MM). **(F)** *Left*: TIM-3 expression in ZA-stimulated BM MM Vγ9Vδ2 T cells without or with αPD1 in the presence of LY294002 (PI3K inhibitor), fludarabine monophosphate (FAMP) (p-STAT1 inhibitor), and the combination thereof. Bars represent the mean values ± SE of 7 experiments. *Right*: cytofluorimetric analysis of TIM-3 expression in ZA-stimulated BM MM Vγ9Vδ2 T cells without or with α-PD-1 and PI3K and/or pSTAT-1 inhibitors from one representative MM.

Next, we looked for possible intersections between the intracellular pathways triggered by αPD-1 and αTIM-3. Previous work from Zhu C. et al. (54) has reported a cross-talk between TIM-3 and PD-1 mediated by the IL-27/pSTAT1/T-bet axis. BM MM Vγ9Vδ2 T cells showed the lowest T-bet (**Figure 6A**), and the highest IL-27R expression (**Figure 6B**). This pattern has recently been reported in severely exhausted T cells from the BM of patients with AML in relapse after allogeneic transplantation (55). αPD-1 treatment did not increase T-bet and/or IL-27R expression in ZA-stimulated BM Vγ9Vδ2 T cells (**Figures 6C, D**). Moreover, low IL-27 levels were detected in the supernatants of BM MM Vγ9Vδ2 T cells which were not modified by αPD-1 (**Figure 6E**).

The PI3K/Akt axis is another intracellular signalling pathway connecting PD-1 and TIM-3 in tumor-infiltrating lymphocytes from patients with head and neck cancer (53). In these cells, TIM-3 up-regulation induced by αPD-1 can be abrogated with LY294002, a broad PI3K inhibitor (53). Thus, we evaluated whether αPD-1-induced TIM-3 up-regulation in BM MM Vγ9Vδ2 T cells could be inhibited by single pSTAT-1 inhibition with fludarabine monophosphate (FAMP) (56), single PI3K inhibition with LY294002, or the combination thereof. Results shown in **Figure 6F** indicate that these pathways are not druggable to prevent αPD-1-induced TIM-3 up-regulation in BM MM Vγ9Vδ2 T cells.

### Improved efficacy by tailoring ICP blockade to the disease status

**Figure 7:**
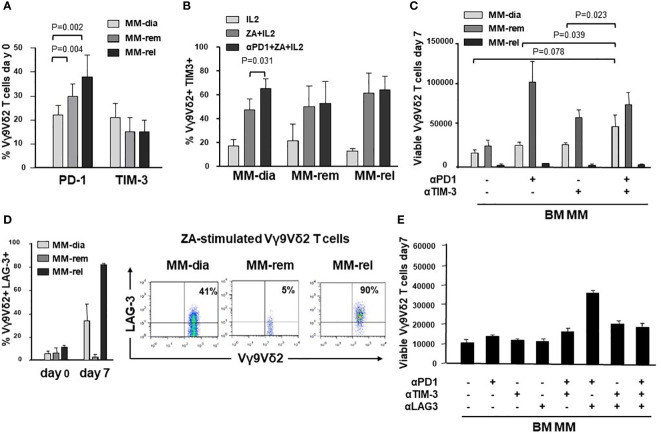
The ICP/ICP-L network is dynamically shaped by the disease status. **(A)** PD-1 and TIM-3 expression in resting BM Vγ9Vδ2 T cells from MM patients at different stages of disease (MM-dia, MM-rem and MM-rel). Bars represent the mean values ± SE from 7 (MM-rel) to 50 (MM-dia). **(B)** TIM-3 expression in BM MM Vγ9Vδ2 T cells after 7-day ZA stimulation in the presence or absence of αPD1. Bars represent the mean values ± SE from 3 (MM-rem) to 6 (MM-dia). **(C)** BM MM Vγ9Vδ2 T-cell proliferation in MM-dia, MM-rem and MM-rel after 7-day ZA stimulation in the presence of αPD1, αTIM-3, and the combination thereof. Bars represent the mean values ± SE from 4 (MM-rem) to 8 (MM-dia). **(D)** *left:* LAG-3 expression in resting (day 0) or ZA-stimulated BM Vγ9Vδ2 T cells from MM patients at different stages of disease (MM-dia, MM-rem and MM-rel). Bars represent the mean ± SE from 4 (MM-rel) to 5 (MM-dia) experiments; *right:* cytofluorimetric analyses of LAG-3 expression in ZA-stimulated Vγ9Vδ2 T cells from one representative MM-dia, MM-rem, and MM-rel. **(E)** BM MM Vγ9Vδ2 T-cell proliferation in MM-rel after 7-day ZA stimulation in the presence αPD1, αTIM-3, and αLAG-3 as single agents or in combination. Bars represent the mean ± SE of 3 experiments.

Next, we investigated whether the ICP/ICP-L immune suppressive circuitry was influenced by the disease status. PD-1 expression was significantly higher in MM-rel than in MM-dia, while MM-rem showed intermediate values. By contrast, no differences were observed in TIM-3 expression between MM-dia, MM-rem, and MM rel (**Figure 7A**). We investigated whether αPD-1 treatment induced TIM-3 up-regulation also in MM-rem and MM-rel. **Figure 7B** shows that TIM-3 was up-regulated in MM-dia only, but not in MM-rem and MM-rel.

The effect of single or dual PD-1/TIM-3 blockade on ZA-induced proliferation in BM MM Vγ9Vδ2 T cells in MM-dia, MM-rem and MM-rel is shown in **Figure 7C**. BM Vγ9Vδ2 T cells from MM-rem were the only ones to reach normal proliferation values with single PD-1 or TIM-3 blockade, the former being slightly more effective than the latter. Dual PD-1/TIM-3 blockade was not superior to single blockade in MM-rem. By contrast dual PD-1/TIM-3 blockade was more effective than single blockade in MM-dia, the only clinical setting in which αPD-1 induces TIM-3 up-regulation. BM Vγ9Vδ2 T cells from MM-rel showed the worst anergy to single and dual blockade, even if TIM-3 expression was similar to MM-dia and MM-rem and was not up-regulated by αPD-1 (**Figures 7A, B**).

These findings prompted us to investigate the expression of additional ICP on BM MM Vγ9Vδ2 T cells in MM-rel. **Figure 7D** shows that LAG-3 expression was similar in resting (day 0) BM Vγ9Vδ2 T cells from MM-dia, MM-rem, and MM-rel. After ZA stimulation, LAG-3 expression was slightly increased in MM-dia, unmodified in MM-rem, and increased in MM-rel, even if the differences was not statistically significant. Next, we determined which PD-1/TIM-3/LAG-3 combination was more effective to mitigate the anergy of BM Vγ9Vδ2 T cells in MM-rel. Results shown in **Figure 7E** indicate that dual PD-1/LAG-3 blockade was more effective than dual PD-1/TIM-3, dual TIM-3/LAG-3, and even triple PD-1/TIM-3/LAG-3 blockade, but still inferior to that reached in MM-rem after single PD-1 or TIM-3 blockade, or MM-dia after dual PD-1/TIM-3 blockade.

These data confirm that the relapse is the most challenging setting, and immune-based strategies should be delivered in remission, when the immune suppressive TME commitment is partially relieved.

## Discussion

In this work, we have used Vγ9Vδ2 T cells as cellular decoders to investigate the role played by the ICP/ICP-L network in the TME of MM patients. A significant proportion of resting BM MM Vγ9Vδ2 T cells showed PD-1 and TIM-3 co-expression, as previously reported in Vγ9Vδ2 T cells chronically exposed to infectious agents (28) or to cancer cells in solid (29, 30) and blood tumors (31). PD-1 and TIM-3 co-expression is considered a phenotypic hallmark of functional exhaustion (24, 26). However, multiple ICP expression is not sufficient per se to identify functionally exhausted cells. One reason is that immune competent T cells can also express ICP after activation, but in this case ICP expression is transient and finalized to dampen T-cell activation to prevent uncontrolled immune reactions and autoimmunity. In contrast, ICP expression on chronically activated T cells reflects a dysfunctional state induced by the long-term exposure to antigens in the context of an inappropriate microenvironment. We have previously shown that BM MM Vγ9Vδ2 T cells are exposed to supra-physiological IPP concentrations released in large amounts by BMSC and, to a lower extent by myeloma cells (57). Thus, BM MM Vγ9Vδ2 T cells fulfil the operational criteria of functionally exhausted cells because: 1) PD-1/TIM-3 co-expression is associated with functional dysfunctions; 2) functional dysfunctions are observed after challenging the normal counterpart (i.e., BM Ctrl Vγ9Vδ2 T cells) with the same antigen (i.e., ZA) in the same microenvironment (i.e., BM) (58). After ZA stimulation, BM MM Vγ9Vδ2 T cells further up-regulated PD-1 and TIM-3 expression. In mice, functionally exhausted cells are hierarchically organized from progenitor to terminally differentiated exhausted T cells (58), the latter being more difficult to rescue than the former. Our data indicate that inadvertent or inappropriate engagement of immune effector cells can worsen functional exhaustion also in humans.

PD-1+ TIM-3+ BM MM Vγ9Vδ2 T cells expressed immune senescence markers (33, 41, 42). Vγ9Vδ2 T cells from normal individuals are particularly resistant to immune senescence due to their peculiar capacity to adapt to life-long stimulation (59). In MM, the immune suppressive TME turns off the capacity of Vγ9Vδ2 T cells to resist life-long stimulation. CD160 expression was mainly restricted to CM and TEMRA BM MM VγVδ2 T cells, which is the subset with the highest ICP expression. Interestingly, the loss of CD27 and CD28 and the expression of TIM-3 and CD57 on T cells has been associated with resistance to ICP blockade (35).

Immune senescence of BM MM Vγ9Vδ2 T cells was confirmed by the expression of pγH2AX. A weak pγH2AX expression was already detectable in freshly isolated BM γδ T cells, but significantly increased after ZA stimulation, whereas no expression was detected in resting or ZA-stimulated BM Ctrl samples. γH2AX phosphorylation is used by mammalian cells to prevent genomic instability after DNA breakage induced by genotoxic stress or senescence (60). Our data indicate that pγH2AX quantification can be used to predict the functional outcome of immune effector cells after stimulation, and not only to screen the genotoxic profile of drugs and to identify senescent cells in aging and disease (61).

The functional plasticity of Vγ9Vδ2 T cells embedded in the immune suppressive TME can lead to the acquisition of regulatory or pro-tumoral functions (46). We have not found any phenotypic or functional evidence to support a regulatory/pro-tumoral shift of BM Vγ9Vδ2 T cells in MM, unlike colon, breast and other solid cancers in which immune senescent γδ T cells have been reported to suppress the proliferation of conventional T cells (62–65).

Exhaustion and immune senescence of BM MM Vγ9Vδ2 T cells were associated with alterations in the TCR signaling pathway. pAKT, pSTAT1, pJAK1, and ZAP-70 were down-regulated, while PTEN was up-regulated in MM BM Vγ9Vδ2 T cells. ZAP-70 was also down-regulated in BM CD3+ Vγ9Vδ2- T cells of MM patients. The significantly lower ZAP-70 expression in BM compared further confirms how powerful is the immune conditioning exerted by the prolonged exposure to tumor cells in the TME. In contrast, we have not observed CD3-ζ chain down-modulation in Vγ9Vδ2 T cells and CD3+ Vγ9Vδ2- T cells unlike previous reports (51). Increasing evidence suggests that ZAP-70 down-regulation in T cells and NK cells can contribute to impairment of anti -tumor immune responses and bias the efficacy of immunotherapy (66). We are currently investigating whether ZAP-70 expression is correlated with Vγ9Vδ2 T-cell dysfunctions in MM.

TIM-3 was significantly up-regulated after ZA stimulation in the presence of αPD1, whereas PD-1 was not up-regulated after ZA stimulation in the presence of αTIM3, indicating a one-way rather than two-way cross-talk between these molecules. TIM-3 up-regulation after PD-1 blockade in conventional T cells is considered a potential mechanism of adaptive resistance to αPD-1 *in vitro* (52, 53, 67) and *in vivo* (52, 53, 68).

Dual PD-1/TIM-3 blockade was more effective than single ICP blockade to partially recover proliferation, IFN-γ production, and CD107 expression in BM Vγ9Vδ2 T cells, and to mitigate the altered expression of TCR-associated molecules. Dual PD-1/TIM-3 blockade has also been reported to up-regulate IFN-γ and TNF-α production in PB Vγ9Vδ2 T cells of AML patients after pAg stimulation (31).

Dual ICP blockade is currently carried on in the clinical setting using mAb combinations willing to improve response rates and/or overcome acquired resistance to single ICP blockade (69). However, this strategy is burdened by clinical and financial toxicities (70), and alternative approaches are under investigation (69, 71). One alternative approach could be the identification of druggable intracellular intersections between these pathways. To this end, we have investigated the IL-27/pSTAT1/T-bet, and the PI3K/AKT pathways that have been reported to connect PD-1 and TIM-3 in tumor-bearing mice and patients with head and neck squamous cell carcinomas (53, 54), but we have not found any evidence of PD-1/TIM-3 cross-talk *via* these pathways in BM MM Vγ9Vδ2 T cells.

Interestingly, T-bet expression was low in resting BM MM Vγ9Vδ2 T cells as recently shown in the BM of patients with AML. In these patients, the emergence of severely exhausted (i.e., T-bet^low^, PD-1+) T cells has been reported to predict disease relapse after allogeneic transplantation (55). By contrast, IL-27R expression was high in BM MM Vγ9Vδ2 T cells, whereas soluble IL-27 levels were low and did not increase after ZA stimulation. We speculate that BM MM Vγ9Vδ2 T cells are equipped with a high number of IL-27R to catch the small amount of IL-27 available in the TME to eventually improve their fitness, and not to up-regulate TIM-3.

This is the first report comparing the role of ICP/ICP-L and their blockade in the TME of MM-dia, MM-rem and MM-rel. PD-1 expression in BM MM Vγ9Vδ2 T cells was significantly higher in MM-rel than in MM-rem and MM-dia, whereas TIM-3 expression was not different. Interestingly, MM-rem showed significantly higher PD-1 expression than MM-dia, indicating that it is not trivial for BM MM Vγ9Vδ2 T cells to get rid of the immune suppressive imprinting operated by the TME. Single or dual blockade PD-1/TIM-3 showed different efficacy according to the disease status. MM-rem showed the best recovery in the presence of the αPD-1 or αTIM-3: the former was slightly better than the latter, whereas the combination did not show any additive or synergistic effect. Dual PD-1/TIM-3 blockade showed an additive effect in MM-dia, whereas MM-rel were totally refractory, no matter single or dual PD-1/TIM-3 blockade was applied. It remains to be determined in MM-rel whether the immune dysfunction anticipates the myeloma cell regrowth or vice-versa.

Our data confirm that the refractory/relapse setting remains the most difficult challenge for immune-based interventions. Paradoxically, this is also the clinical setting usually selected for first-in-man or phase I/II studies, including MM (72), with the risk to jeopardize future investigation since results will rarely meet clinical expectations. Interestingly, BM Vγ9Vδ2 T cells from MM-rel significantly up-regulated LAG-3 after ZA stimulation in addition to PD-1 and TIM-3. In the MC38 mouse tumor model, dual PD-1/TIM-3 blockade increases the expression of LAG-3 in T cells, and LAG-3 expression confers resistance to αPD-1/αTIM-3 treatment (73). Increased LAG-3 expression in T cells of patients with non-small cell lung cancer (NSCLC) has been associated with resistance to αPD-1 treatment and shorter progression-free survival (22). Likewise, co-expression of PD-1, TIM-3, and LAG-3 in TILs of patients clear cell renal cell carcinoma (CCRC) has been associated with high risk of early progression (23).

Dual PD-1/LAG-3 blockade was the most effective combination to improve the proliferative responses to ZA stimulation in MM-rel, confirming the profound immune suppressive TME commitment in this setting. Triple PD-1/TIM-3/LAG-3 blockade has been proposed to overcome this barrier in syngeneic mouse tumor models (73), but in our hands triple blockade was less effective than dual PD-1/LAG-3 blockade. Alternative strategies can be dual ICP blockade after lymphodepletion by whole body radiation, as reported in the 5T33 murine MM model (74), or after the addition of TGF-β inhibitors as reported by Kwon et al. (25), but these strategies are not easy to apply to humans.

In conclusion, the immune suppressive TME contexture in MM is under dynamic evolution and ICP blockade should be individually tailored to gain the maximum efficacy. The remission phase remains the most favorable setting to deliver Vγ9Vδ2 T-cell-based immune interventions.

## Data availability statement

The original contributions presented in the study are included in the article/**Supplementary material**. Further inquiries can be directed to the corresponding author.

## Ethics statement

The studies involving human participants were reviewed and approved by Comitato Etico Interaziendale A.O. Santa Croce e Carle di Cuneo AA. SS. LL. Cuneo 1, Cuneo 2, Asti. n.176-19 December 11, 2019. The patients/participants provided their written informed consent to participate in this study.

## Author contributions

CG, BC, and JK performed the experiments, analyzed the data, and contributed to the manuscript writing and editing; MM and CR designed and supervised the experiments, analyzed the data and wrote the manuscript; ET, IA, MDA, and AL managed samples collection, analyzed and correlated clinical data, and contributed to the manuscript editing. All authors contributed to the article and approved the submitted version.
