# Supplementary material for: Immune dysfunctions affecting bone marrow Vγ9Vδ2 T cells in multiple myeloma: Role of immune checkpoints and disease status
Source: Front Immunol. 2022 Dec 20;13:1073227. doi: 10.3389/fimmu.2022.1073227 (PMC9808386; doi:10.3389/fimmu.2022.1073227)
Supplement: Supplementary file 1 [file DataSheet_1.docx]

**SUPPLEMENTAL DATA**

**Immune dysfunctions affecting bone marrow Vγ9Vδ2 T cells in multiple myeloma: role of immune checkpoints and disease status**

**Running title:** MM ICP/ICP-L network revealed by Vγ9Vδ2 T cells

Claudia Giannotta^1^†, Barbara Castella^1, 2^†, Ezio Tripoli^1^, Daniele Grimaldi^2^, Ilaria Avonto^3^, Mattia D’Agostino^4^, Alessandra Larocca^4^, Joanna Kopecka^5^, Mariella Grasso^2^, Chiara Rig^5^, and Massimo Massaia^1,2*^

| **Supplemental Table 1**. *List of antibodies used for flow cytometry* | | | |  |
| --- | --- | --- | --- | --- |
| **Antigen** | **Fluorochrome** | **Catalog #** | **Company** | **clone** |
| TCR Vγ9 | FITC | 130-125-242 | Miltenyi Biotech | REA470 |
| TCR Vγ9 | PE | 130-107-434 | Miltenyi Biotech | REA470 |
| TCR Vγ9 | APC | 130-111-010 | Miltenyi Biotech | REA470 |
| CD3 | PerCpC-Vio700 | 130-113-141 | Milteny Biotech | REA613 |
| Program death-1 (PD-1) | APC | 130-120-383 | Milteny Biotech | REA1165 |
| T-cell immunoglobulin mucin 3 (TIM-3) | PE | 130-117-364 | Milteny Biotech | F38-2E2 |
| Lymphocyte-activation gene 3 (LAG-3) | PE | 369306 | BioLegend | [11C3C65](https://www.biolegend.com/en-us/search-results?Clone=11C3C65) |
| Cytotoxic T-Lymphocyte Antigen 4 (CTLA-4) | APC | [130-124-016](https://www.miltenyibiotec.com/IT-en/products/cd152-antibody-anti-human-bni3.html?utm_source=3rd_biocompare&utm_medium=product_listing&utm_campaign=4_Recombinant_antibodies_for_smarter_analysis#copy-to-clipboard) | Miltenyi Biotech | BNI3 |
| CD27 | FITC | 130-113-639 | Miltenyi Biotech | REA499 |
| CD45RA | PerCP | 304156 | BioLegend | HI100 |
| CD57 | FITC | 130-122-935 | Miltenyi Biotech | TB03 |
| CD28 | PE | [130-126-202](https://www.miltenyibiotec.com/IT-en/products/cd28-antibody-anti-human-15e8.html#copy-to-clipboard) | Miltenyi Biotech | 1,50E+09 |
| CD160 | APC | 341208 | BioLegend | [BY55](https://www.biolegend.com/en-us/search-results?Clone=BY55) |
| CD39 | PE | 130-110-650 | Miltenyi Biotech | REA739 |
| CD38 | FITC | 356610 | Biolgend | HB-7 |
| CD73 | PE | 130-112-060 | Miltenyi Biotech | REA804 |
| CD4 | PerCP-Vio 700 | 130-113-228 | Miltenyi Biotech | REA623 |
| CD8 | APC | [130-113-154](https://www.miltenyibiotec.com/IT-en/products/cd8-antibody-anti-human-bw135-80.html#copy-to-clipboard) | Miltenyi Biotech | BW135/80 |
| Programmed Death-Ligand 1 (PD-L1) | PE | 329706 | BioLegend | 29E.2A3 |
| Galectin-9 (Gal-9) | APC | 130-106-474 | Miltenyi Biotech | REA435 |
| IL-17A | PE | 12-7179-42 | eBioscience | eBio64DEC17 |
| ZAP-70 | PE | 313404 | BioLegend | 1E7.2 |
| CD3-ζ chain | FITC | 644103 | Biolegend | 6B10.2 |
| IFNγ | APC | 502512 | BioLegend | 4S.B3 |
| CD107 | PE | 328608 | BioLegend | H4A3 |
| Tbet | APC | 130-098-607 | Miltenyi Biotech | REA102 |
| IL27 Receptor (R-IL27) | APC | FAB14791A | R&D Systems | 191106 |
|  |  |  |  |  |
|  |  |  |  |  |
|  |  |  |  |  |
|  |  |  |  |  |

| **Supplemental Table 2**. *List of antibodies used for Western Blot* | | |  |  |
| --- | --- | --- | --- | --- |
| **Antibody name** | **clone** | **Diluition** | **Catalog #** | **Company** |
| anti-phopho(Ser139)γ-H2A.X | rabbit polyclonal | 1:1000 | ab11171 | Abcam, Cambridge, MA |
| phospho(Ser473)Akt | rabbit polyclonal | 1:1000 | 9271 | Cell Signallling Technology, Danvers, MA |
| anti-Akt | rabbit polyclonal | 1:1000 | 9272 | Cell Signallling Technology, |
| anti-SHP2 | rabbit polyclonal | 1:500 | 131541 | Abcam |
| anti-PTEN | rabbit polyclonal | 1:500 | 137337 | Abcam |
| anti-phospho(Tyr1022/1023) JAK1 | rabbit clone 59H4L5 | 1:1000 | 700028 | ThermoFisher Scientific, Waltham, MA |
| anti-JAK1 | rabbit clone JM75-03 | 1:2000 | MA5-32780 | ThermoFisher Scientific, Waltham, MA |
| anti-phospho(Tyr701)-STAT1 | rabbit clone 15H13H67 | 1:1000 | 700349 | ThermoFisher Scientific, Waltham, MA |
| anti-STAT1 | mouse clone STAT1-79 | 1:500 | HAO0832 | ThermoFisher Scientific |
| β-tubulin | mouse clone D10 | 1:500 | sc5274 | Santa Cruz Biotechnology, Santa Cruz, CA; |

**Supplemental Figure 1**


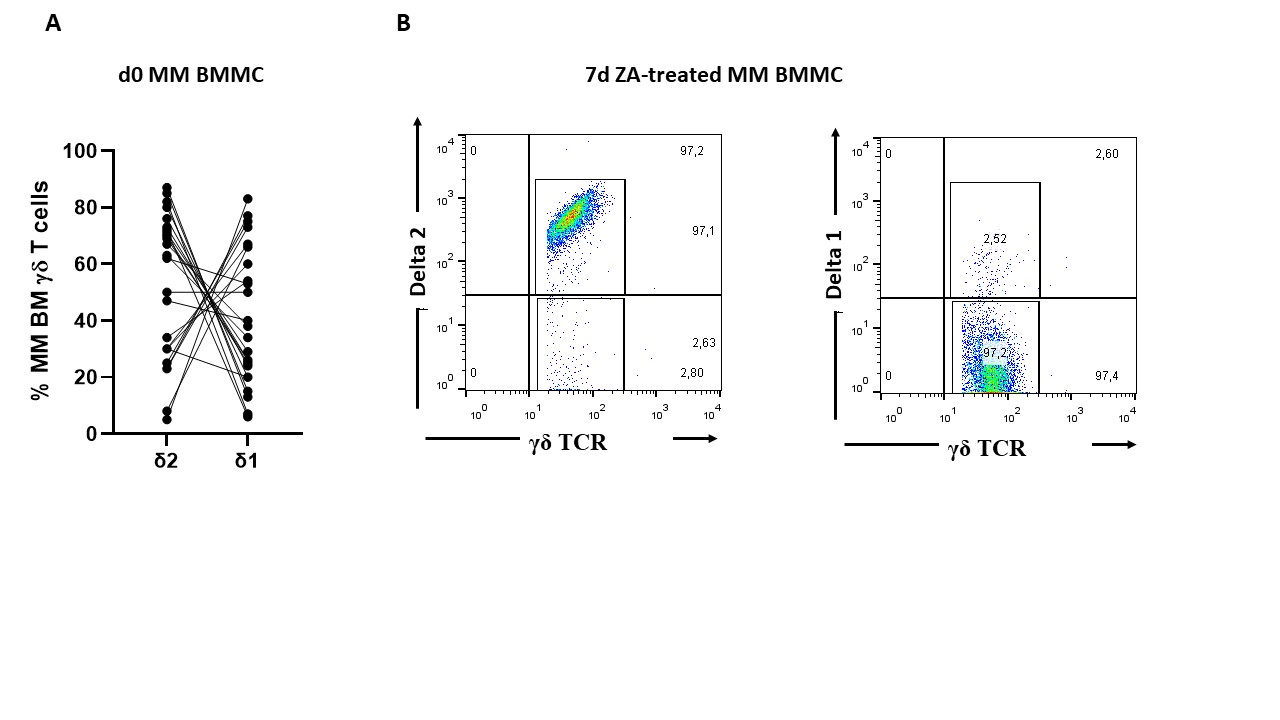


**Vδ1 and Vδ2 T-cell subset distribution. A)** Vδ1 and Vγ9Vδ2 T cells (Vδ2) distribution in freshly isolated (day 0) BMMC from 24 MM-dia; **B)** Representative cytofluorometric analyses of Vδ1 and Vγ9Vδ2 T cell distribution after 7day stimulation of BMMC with ZA. Based on these data, we postulate that isolation of γδ T cells by immune magnetic separation using Anti-pan-γδ-conjugated magnetic microbeads (Miltenyi Biotec, Germany #130-050-701) will result in mixed Vδ1/Vδ2 subpopulations in freshly isolated BMMC (day 0) and almost only Vγ9Vδ2 T cells after ZA stimulation which is specific for these cells.

**Supplemental Figure 2**


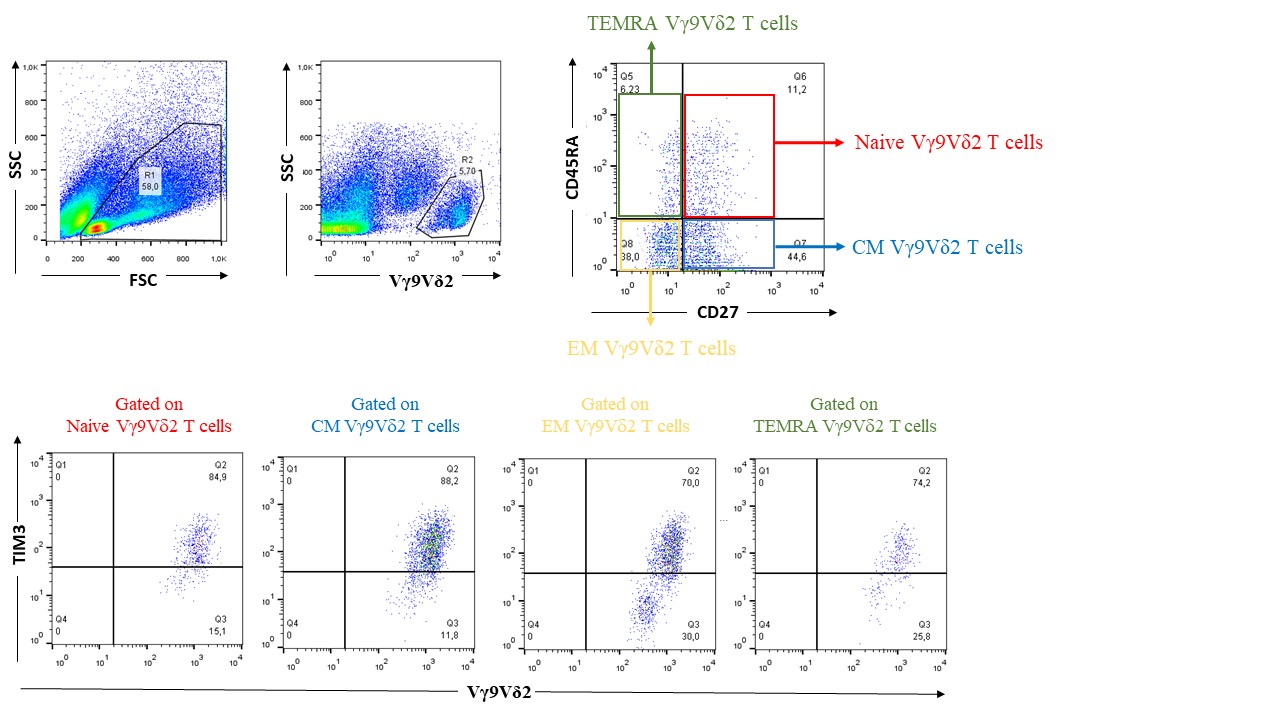


**TIM-3 expression in Vγ9Vδ2 T-cell subsets .** Gating strategy used to investigate TIM-3 expression Vγ9Vδ2 T-cell subsets..

**Supplemental Figure 3**


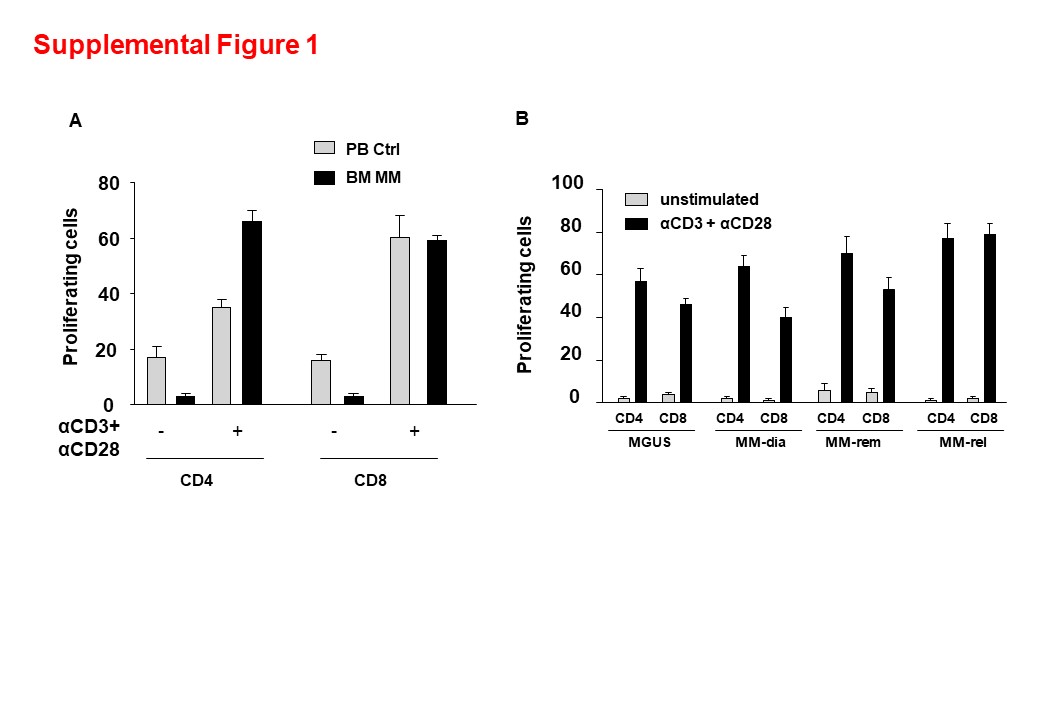


**Preserved proliferation of BM MM CD4+ and CD8+ T cells to αCD3+αCD28 stimulation**

**A)** CFSE-based proliferation of PB Ctrl and BM MM CD4+ and CD8+ T cells after 72 hours stimulation with αCD3+αCD28 stimulation. Proliferative responses are similar to those of PB Ctrl CD4+ and CD8+ cells. Bars represent mean values ± SE from 5 (PB Ctrl) to 10 (BM MM) experiments. Differences are not statistically significant **B)** CFSE-based proliferation of BM MM CD4+ and CD8+ T cells from MGUS and MM at different stages of the disease (MM-dia; MM-rem; MM-rel) after 72-hour stimulation with αCD3 + αCD28. BM MM CD4+ and CD8+ cell proliferation are not affected by the disease status. Bars represent mean values ± SE from 3 (MGUS, MM-rem, MM-rel) to 4 (MM-dia) experiments.

**Supplemental Figure 4**


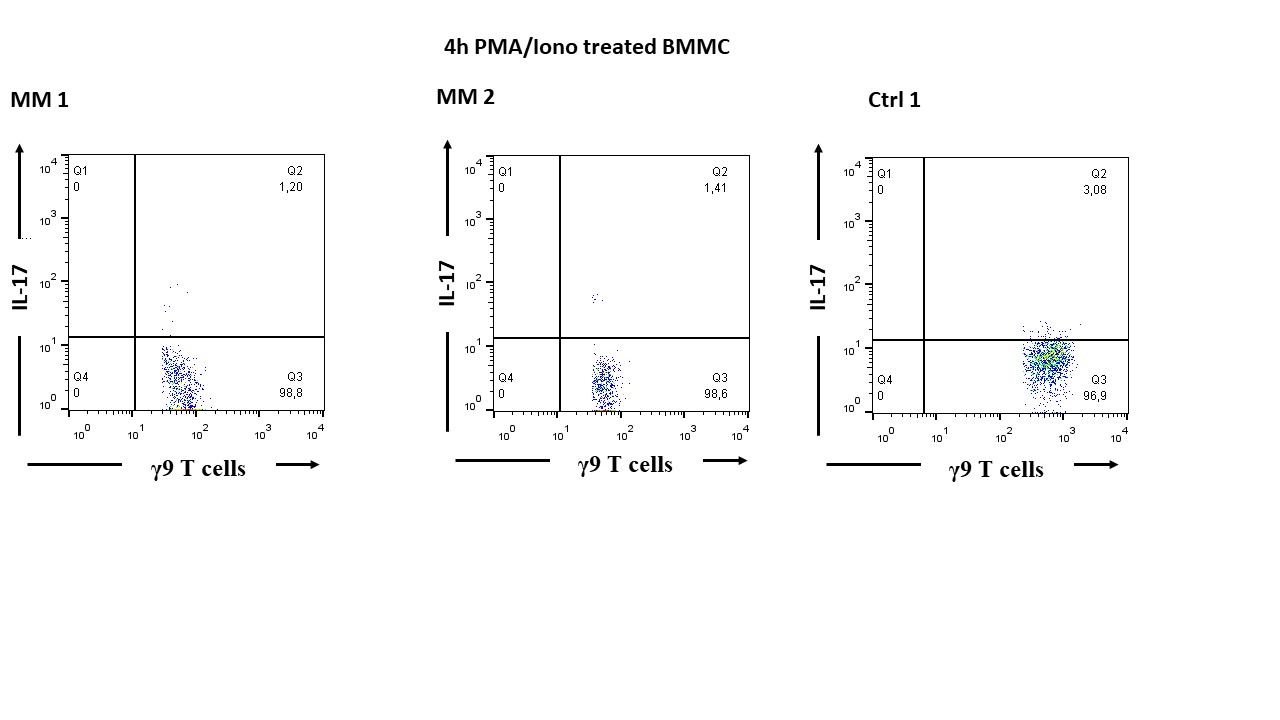


**IL-17 expression in BM MM and Ctrl Vγ9Vδ2 T cells.** IL-17 production was evaluated in freshly isolated BMMC after 4-hour incubation at 37°C with PMA (50 ng/ml)+ ionomycin (1 μg/ml) followed by 1-hour incubation with brefeldin (500 ng/ml).

**Supplemental Figure 5**


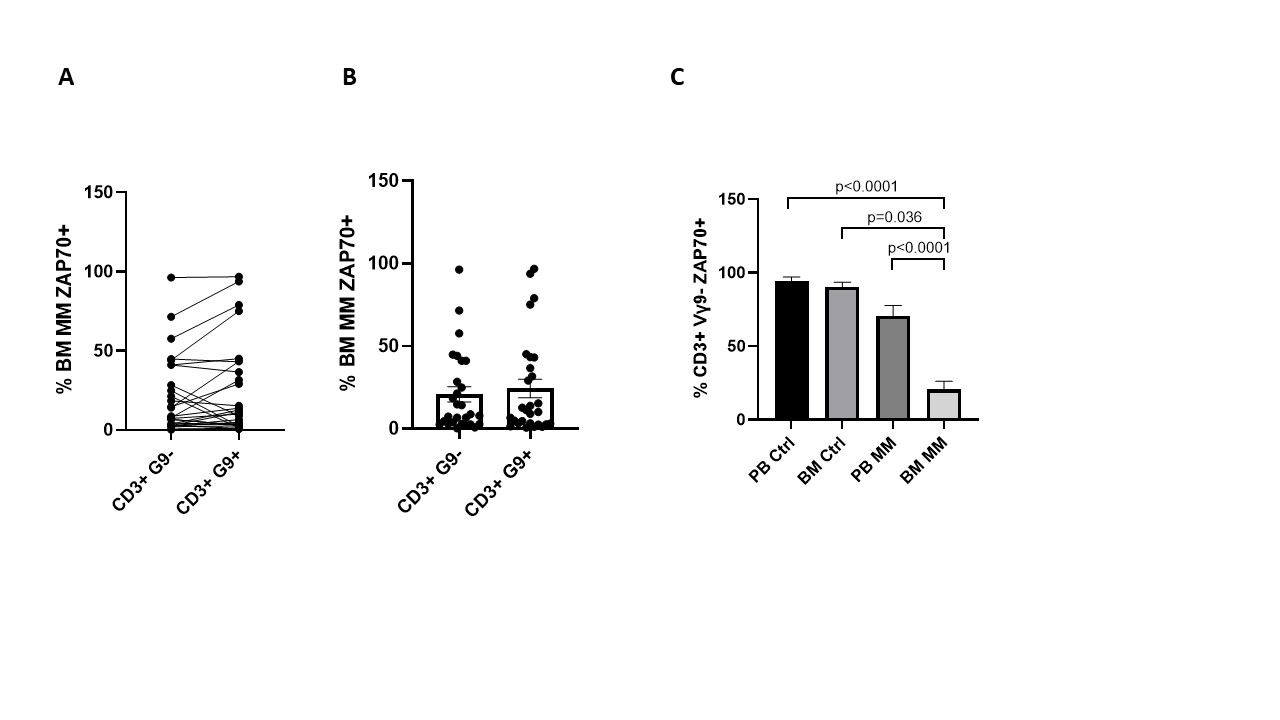


**Analysis of ZAP-70 expression in Vγ9Vδ2 and CD3+ Vγ9Vδ2^neg^ cells. A**) Paired analysis of ZAP-70 expression in Vγ9Vδ2 (CD3+ G9+) and CD3+ Vγ9Vδ2^neg^ (CD3+ G9-) cells from 28 BM MM-dia. **B**) Resuls are shown as bars representing mean values ± SE of 28 experiments. The differences is not statistically significant. **C**) ZAP-70 expression in freshly isolated PB and BM CD3+ Vγ9Vδ2^neg^ T cells from Ctrl and MM-dia. BM MM Vγ9Vδ2 T cells have the lowest mean ZAP-70 expression. Bars represent mean values ± SE from 3 (BM Ctrl) to 14 (PB MM) experiments.

**Supplemental Figure 6**


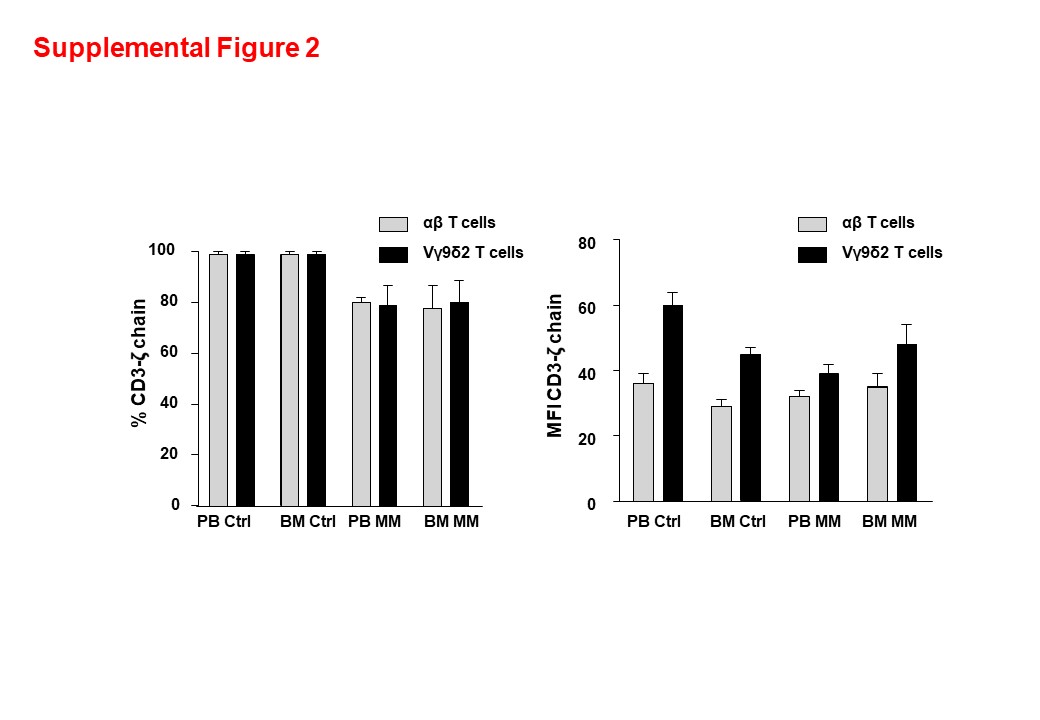


**Preserved CD3-ζ chain expression in BM MM Vγ9Vδ2 T cells.** Percentage (left) and MFI (right) of CD3-ζ chain expression are similar in PB and BM Ctrl and MM Vγ9Vδ2 T cells. Bars represent mean values ± SE from 3 (PB Ctrl, BM Ctrl and PB MM) to 14 (BM MM) experiments.

**Supplemental Figure 7**


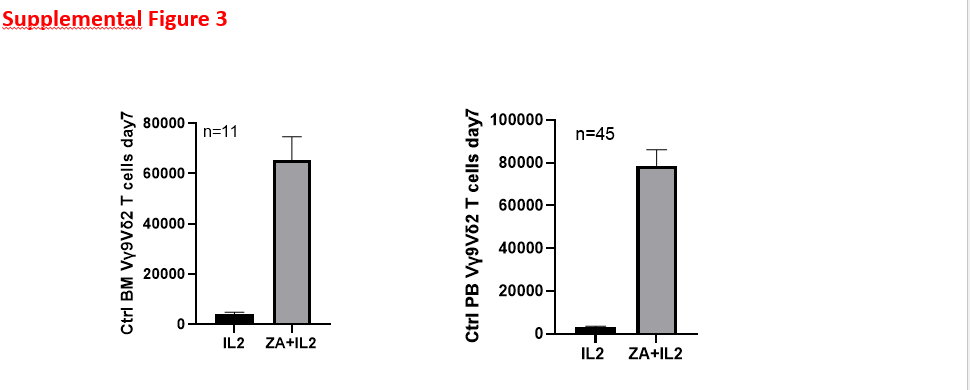


**Proliferative response of Ctrl PB and BM Vγ9Vδ2 T cells after ZA stimulation.** Bars represents mean values ± SE of 11 experiments in BM Ctrl (left) and 45 experiments in PB Ctrl (right). Viable Vγ9Vδ2 T cells were counted on day 7 with the trypan blue staining assay and flow cytometry after gating for CD3 in combination with αVg9 mAb. Numerical values are: BM Ctrl: 62,187±10,562; PB Ctrl: 78,676 ± 7,486

|  |
| --- |
|  |

**Supplemental Figure 8**


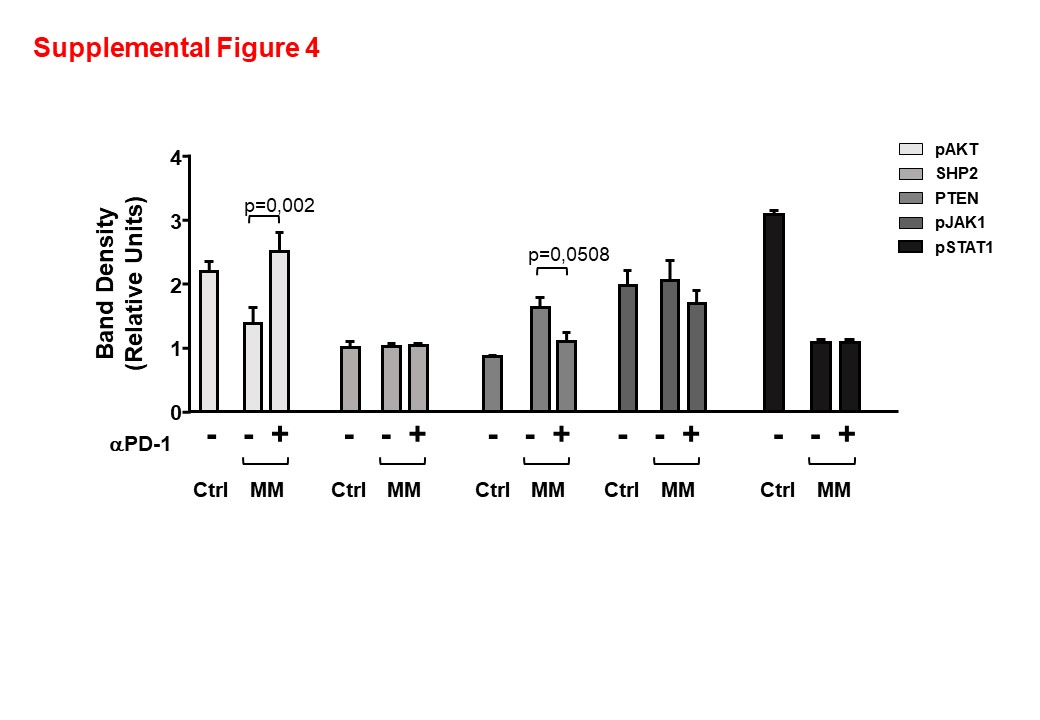


**αPD-1 effect on TCR-associated molecules in ZA-stimulated BM MM ~~Vγ9Vδ2~~ γδ T cells**.

Densitometric analysis of pAKT, AKT, SHP2, PTEN, pJAK-1, JAK-1, pSTAT-1, and STAT-1 expression in BM MM Vγ9Vδ2 T cells after ZA stimulation in the absence or in the presence of αPD1 Bars represent mean values ± SE from 3 (BM Ctrl) to 10 experiments (BM MM).
